# Gene models from ESTs (GeneModelEST): an application on the *Solanum lycopersicum *genome

**DOI:** 10.1186/1471-2105-8-S1-S9

**Published:** 2007-03-08

**Authors:** Nunzio D'Agostino, Alessandra Traini, Luigi Frusciante, Maria Luisa Chiusano

**Affiliations:** 1 Department of Structural and Functional Biology, University 'Federico II', 80126 Naples, Italy; 2 Department of Soil, Plant, and Environmental Sciences, University 'Federico II', 80055 Portici, Naples, Italy

## Abstract

**Background:**

The structure annotation of a genome is based either on *ab initio *methodologies or on similaritiy searches versus molecules that have been already annotated. *Ab initio *gene predictions in a genome are based on *a priori* knowledge of species-specific features of genes. The training of *ab initio *gene finders is based on the definition of a data-set of gene models. To accomplish this task the common approach is to align species-specific full length cDNA and EST sequences along the genomic sequences in order to define exon/intron structure of mRNA coding genes.

**Results:**

GeneModelEST is the software here proposed for defining a data-set of candidate gene models using exclusively evidence derived from cDNA/EST sequences.

GeneModelEST requires the genome coordinates of the spliced-alignments of ESTs and of contigs (tentative consensus sequences) generated by an EST clustering/assembling procedure to be formatted in a General Feature Format (GFF) standard file. Moreover, the alignments of the contigs versus a protein database are required as an NCBI BLAST formatted report file.

The GeneModelEST analysis aims to i) evaluate each exon as defined from contig spliced alignments onto the genome sequence; ii) classify the contigs according to quality levels in order to select candidate gene models; iii) assign to the candidate gene models preliminary functional annotations.

We discuss the application of the proposed methodology to build a data-set of gene models of *Solanum lycopersicum*, whose genome sequencing is an ongoing effort by the International Tomato Genome Sequencing Consortium.

**Conclusion:**

The contig classification procedure used by GeneModelEST supports the detection of candidate gene models, the identification of potential alternative transcripts and it is useful to filter out ambiguous information. An automated procedure, such as the one proposed here, is fundamental to support large scale analysis in order to provide species-specific gene models, that could be useful as a training data-set for *ab initio *gene finders and/or as a reference gene list for a human curated annotation.

## Background

Genome annotation involves as a primary task the identification of gene locations and the definition of gene structures along DNA sequences. This is carried out mainly via computational approaches. Several *in silico *methods can be used for gene annotation in a genome [1-3]. These methods can be based on *ab initio *predictions or on similarity searches. The *ab initio *gene finder tools attempt to recognise genes within genomic sequences, locating regions with a coding potential and detecting signals known as typical of gene structures, such as promoters, termination signals, splice junction boundaries (acceptor/donor) etc. [4]. *Ab initio *methods need to be trained on a set of known genes assuming genes within a genome share similar compositional properties which are typical of the species. The training data-set should be composed by gene models of which the structure is experimentally defined. Currently, the most valuable method for the definition of the structure of mRNA coding genes relies on full length cDNA sequences and on their spliced-alignment versus the DNA sequence [5-9]. However, full length cDNA sequencing is an expensive and time-consuming approach when compared to the high-throughput EST sequencing. Then, though EST intrinsic shortcomings due to contaminations and limited sequence quality, these data represents a valuable source of information to accomplish the task of gene model building [10].

There are genome-based clustering methodologies to assemble ESTs into one gene [11]. Other approaches are based on the definition of genome-independent clusters.  Each cluster, which is created by grouping overlapping EST, corresponds to a unique gene.  A cluster can consist of one or multiple contigs.  In this last case ESTs may yield evidence for the existence of alternative transcripts or splicing isoforms [12,13].  Contigs are assembled in the attempt to cover the whole sequence of an mRNA [14,15]. Therefore, the alignment of a contig versus the genomic sequence may represent a partial or a complete exon/intron structure of a gene.. In the absence of errors, ESTs and contigs can be completely aligned to the corresponding DNA sequence (EST/contig to genome mapping) using specialized algorithms [16,19].

We present here GeneModelEST, a software to assess the reliability of contigs in order to define gene models. The approach is based on the availability of spliced-alignments of the contigs onto a genomic sequence and on the evaluation of pairwise comparisons of each exon candidate defined from a contig versus the EST data, which are independently aligned onto the same genomic sequence.

The software was implemented to define a reliable and a consistent number of species-specific gene models using a rigorous approach. In fact, each gene model is strictly supported by experimental evidence and lacks possible ambiguities that could cause both inconsistencies in the definition of the gene properties and a sidetracked training of gene finders.

We discuss the application of the proposed methodology to build a data-set of gene models of *Solanum lycopersicum*, whose genome sequencing is an ongoing effort by the International Tomato Genome Sequencing Consortium [20]. In this preliminary phase of the project, a reference set of gene models is still necessary to train software for gene prediction and to investigate on relevant gene features in this species.

## Methods

### Input file format

Two GFF (General Feature Format) [21] formatted files are required as input for GeneModelEST. They report the *in silico *derived coordinates of contigs and ESTs to genome alignments.

An NCBI BLAST formatted report file, describing the alignments of the contigs versus a protein database, is required too.

The GFF files must include two features:

1. the 'match' feature: indicating the start and the end positions that span the entire genomic region which resulted aligned versus the corresponding EST/contig

2. the 'HSP' (High-Scoring Pairs) feature: indicating the start and the end positions of the local alignments of the genomic region versus the EST/contig. The 'HSPs' describe all the consecutive elements which correspond to exons in a 'match' region on the genome sequence. The regions of the genomic sequence that span two consecutive HSPs correspond to intron regions.

### Evaluation of exon candidates

**Figure 1:**
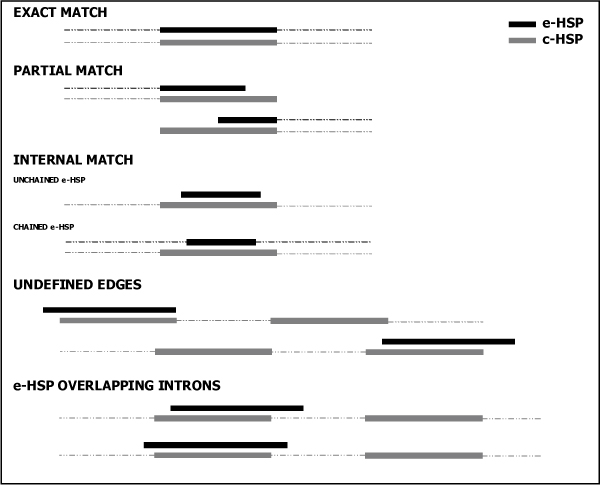
Representation of the possible instances resulting from pairwise comparisons of c-HSPs and e-HSPs: i) **exact match: **the start and end positions of the e-HSP coincide with those of the c-HSP; ii) **partial match: **the e-HSP and c-HSP share one of the edges and the other edge of the e-HSP is included in the c-HSP region; iii) **internal match: **an e-HSP lies within a c-HSP. Internal matches can be distinguished in two cases: a) *unchained e-HSP*: in case the e-HSP is completely included in the corresponding c-HSP or b) *chained e-HSP*: in case the e-HSP is concatenated to surrounding e-HSPs; iv) **undefined edges**: an e-HSP is overlapping a terminal c-HSP beyond one of the contig boundaries; v) **e-HSP overlapping introns**: if one or both the edges of an e-HSP lie on an intronic region of the contig.

The first step of the GeneModelEST analysis considers the coordinates of the 'match' feature of the contigs in order to define the contig-to-contig status, as follows:

1) contigs sharing overlapping regions along a genome sequence.

2) contigs with no overlaps;

The second step evaluates exons (c-HSP) of not-overlapping contigs through pairwise comparisons versus all the 'HSP' features belonging to the EST sequences (e-HSP) which have been aligned in the same region. Possible results of the pairwise comparisons are classified according to the following instances (Figure 1):

i) exact match: the start and the end positions of an e-HSP coincide with those of the c-HSP;

ii) partial match: at least one of the edges of the e-HSP is coinciding with one of the edges of the c-HSP. Therefore the other edge of the e-HSP is included in the region spanned by the c-HSP because of EST length-limit.

iii) internal match: the start and the end positions of the e-HSP are included in the corresponding c-HSP. This instance can be split in two cases according to the e-HSP status:

a) chained e-HSP: the e-HSP is included in the c-HSP and it is concatenated with surrounding e-HSPs. Therefore the e-HSPs and its flanking intronic regions are overlapping the c-HSP. This indicates that the EST, which the e-HSP belongs to, has an exon shorter than the corresponding one in the contig;

b) unchained e-HSP: the e-HSP is included in the corresponding c-HSP and it is not concatenated with other surrounding e-HSPs.

iv) undefined edges: the e-HSP is overlapping a terminal c-HSP beyond one of the contig boundaries. This implies that the EST, which the e-HSP belongs to, is describing a terminal exon longer than the one defined by the contig under consideration. This could happen in case of two possible alternative transcripts or in case of a contig describing only part of the mRNA molecule.

v) e-HSP overlapping introns: one or both the edges of an internal e-HSP lie with an intronic region of the contig. This implies that the EST, which the e-HSP belongs to, is representing an intron retaining sequence, or an alternatively spliced transcript of the same gene, or the transcript of a gene overlapping the same locus.

The status of each c-HSP is defined as *confirmed*, *undefined *or *ambiguous*, according to the possible combinations of the delineated instances as described in Table 1.

### Evaluation of contig quality

A contig can be classified, according to the evaluation of all its c-HSPs, into one of three different classes: *optimal*, *acceptable *and *rejected*. *Optimal *contigs have all the c-HSPs *confirmed*; *acceptable *contigs have at least one c-HSP classified as *undefined*; *rejected *contigs have at least one *ambiguous *c-HSP. Indeed, possible alternative exons, such as those described either by the presence of at least one *chained e-HSP *or by the occurrence of at least one *e-HSP overlapping introns*, indicate alternative gene structure organizations.

Alternative gene structures must be avoided in the definition of candidate gene models. Therefore, GeneModelEST declares as candidate gene models those contigs classified as *optimal *or *acceptable*. *Rejected* contigs are excluded from the automated definition of candidate gene models because they represent either possible alternative splicing or intron retaining sequences, and therefore, they need a human curated validation.

### Contig functional classification

In order to define a functional annotation of the contigs, GeneModelEST evaluates the protein sequence coverage (%coverage) and the similarity threshold (%positives) of the highest scoring alignments as described in the NCBI BLAST report formatted file to define a specific class:

1. *Complete *contigs (coverage ≥ 95%), classified as:

a. *identical to *(similarity ≥ 90%)

b. *similar to *(similarity < 90%);

2. *Uncomplete *contigs (50% ≤ coverage < 95%), classified as:

a. *similar to *(similarity ≥ 60%)

b. *low similarity to *(similarity < 60%).

3. *Undefined product: *a contig with a coverage of the protein < 50%.

4. *Expressed product: *a contig without BLAST matches.

### Output files

GeneModelEST gives four different output files:

1. A CSV (Comma Separated Values) formatted file describing the contig-to-contig status in case of overlapping contigs.

2. A CSV formatted file listing each contig, its identifier, its classification status and its functional class.

3. and 4. Two GFF formatted files, reporting the coordinates of the contigs classified as candidate gene models, compatible with the Gbrowse [22] and with the Apollo [23] software, respectively. This is because the Gbrowse and the Apollo software are considered some of the most referenced software to view and to manually curate gene models.

## Results

GeneModelEST has been used to build a dataset of gene models for *Solanum lycopersicum*. The *S. lycopersicum *genome sequencing is an ongoing project and in this preliminary phase a reference set of gene models is necessary to train software for gene predictions and to explore the gene features in this species. The spliced-alignments of EST/contig data to genome sequences are fundamental for the characterization of gene structures and for the annotation of mRNA coding regions [10,24,25] even in absence of trained gene finders.

200,438 EST sequences of *S. lycopersicum *were downloaded from the dbEST division of GenBank (release 010206). All the ESTs as well as the 16,888 contigs, derived from the EST clustering/assembling procedure and available in the TomatEST database [26], were independently aligned using the blEST software [15] versus the 163 genomic sequences released as BAC contigs in the October 2006 by the Tomato Genome Sequencing Consortium. The results were converted into GFF formatted files, using an in-house implemented Perl script. 1,444 contigs and 16,397 ESTs resulted aligned versus the BAC sequences.

All the contig sequences were also compared versus the UniProt database [27] (release March 2006), setting an E-value < 0.001 as a significant threshold, in order to provide an NCBI multi-BLAST formatted report file.

The proposed methodology supports the classification of contigs that overlap in the same genome region. Indeed the overlapping contigs may represent alternative transcribed forms of the same gene or overlapping genes in a genomic region. However, they are not considered by GeneModelEST in subsequent validations, because they represent ambiguities in the definition of a unique gene model in that genome region.

The analysis of the contig exon candidates concerning the 1,101 not overlapping contigs has produced the following results: 2,762 *confirmed *c-HSPs supported by 13,538 e-HSPs; 816 *undefined *c-HSPs, supported by 7,942 e-HSPs; 355 *ambiguous *c-HSPs, supported by 28,344 e-HSPs.

**Figure 2:**
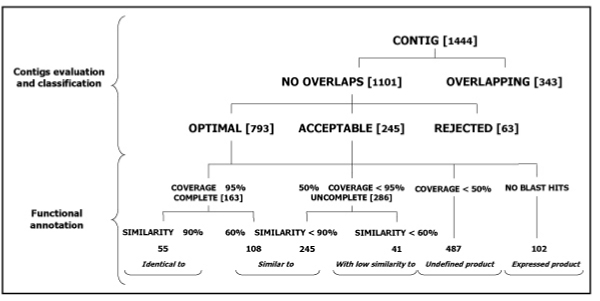
Contig validation procedure: classification and results from the analysis of 163 BAC sequences from *S. lycopersicum*.

Considering the data mapped onto the 163 BAC sequences, GeneModelEST defined 793 *optimal*, 245 *acceptable *and 63 *rejected *contigs (Figure 2). Let us consider the entire data-set of candidate gene models, made up by the *optimal *and the *acceptable *contigs: there are 163 *complete *gene models which cover at least the 95% of the length of the most similar protein sequence in the UniProt database. Among those ones, only 55 gene models have been classified as *identical to *the matching protein and 108 have been instead classified as *similar to *(Figure 2). Among the 286 *uncomplete *gene models, that are those not showing a complete coverage of the protein in the database (50% ≤ coverage < 95%), 245 have been classified as *similar to *(similarity ≥ 60%) the matching protein, while the remaining 41 gene models have been classified *with low similarity to *(similarity < 60%).

487 gene models have been classified as *undefined products *(protein coverage < 50%), while 102 gene models have been classified as *expressed products*, because they do not have BLAST hits.

## Discussion

The need of defining experimentally validated gene structures from an organism is fundamental to get reliable knowledge on compositional properties of the genes and to provide a reference set of species-specific genes suitable to train gene finders.

The availability of a vast amount of EST sequences represents a valuable source to properly confirm the quality of the predicted protein-coding genes in a genome and to define a consistent number of reliable gene models in case no sufficient information is yet available.

In theory, a manual annotation [23] made by experts, who examine both data from experimental results and from computational predictions, should produce the most reliable set of gene models.

In practice, the vast amount of data to analyse represents a drawback for the human annotation. Moreover, there may be conflicts due to the reliability of the predictions, to the quality of the data, to the algorithms used to produce spliced-alignments onto the genome sequence.

GeneModelEST has been designed to define a rigorous set of gene models, strictly supported by experimental evidence. GeneModelEST filters out possible ambiguities that could cause as inconsistencies in the definition of species-specific gene properties as a sidetracked training of gene finders. The software is based on the evaluation of contigs that are derived from EST-based clustering/assembling procedures and that are aligned to the corresponding genome sequences. EST sequences independently mapped onto the genome sequence provide the reference that supports the evaluation of the exons that the contig describes along the genome sequence.

The methodology separates contigs that overlap the same genomic regions from those that are positioned alone along the genomic region.

Contigs with all the exons univocally confirmed by EST evidence are considered optimal gene models.

The similarity based functional annotation of the contigs is used as a reference in the attempt to automatically define those contigs that are putatively complete/uncomplete and sharing/not sharing similarities (with pre-defined settings) to proteins as described in the NCBI BLAST formatted report file. However, one must be aware of the fact that the preliminary functional annotation based on this approach provides just an indicative idea of the real completeness as well as of the functionality of the gene.

GeneModelEST output is suitable for deeper analysis from manual curators, indeed an Apollo compatible GFF format output is provided. The Gbrowse compatible GFF output is provided to support immediate integration of the gene models in a Gbrowse based platform.

The proposed methodology is also useful to further validate previously defined genes on the basis of novel EST sequence data-sets.

## Availability of gene models for *Solanum lycopersicum*

The *in silico *derived coordinates of candidate gene models obtained from *S. lycopersicum *using GeneModelEST are accessible through the graphical annotation viewer Gbrowse [22] at [28]. Optimal and acceptable contigs and their functional annotation are reported.

## Availability and requirements

The software is freely available for no profit users upon request. It requires a Perl interpreter version greater than 5.8.*.

## Authors' contributions

N. D'Agostino and A. Traini contributed equally to this work.

## Figures and Tables

**Table 1:** c-HSP status definition.

**cHSP status**	**exact match**		**partial match**		**internal match**				**undefined edges**		**e-HSP overlapping introns**
					Unchained e-HSP		Chained e-HSP				
	
**Confirmed**	X	**OR**	X	**OR**	X	**AND**	0	**AND**	0	**AND**	0
**Undefined**	X	**OR**	X	**OR**	X	**AND**	0	**AND**	X	**AND**	0
**Ambiguous**	X	**OR**	X	**OR**	X	**AND**	X	**OR**	X	**AND**	X

The definition of the status of a c-HSP is the result of all the pairwise comparisons versus e-HSP aligned in the genomic region. X indicates at least one occurrence of the corresponding instance. To define the c-HSP status Boolean operators have been used.
